# Magnesium Isoglycyrrhizinate Ameliorates Concanavalin A-Induced Liver Injury by Inhibiting Autophagy

**DOI:** 10.3389/fphar.2021.794319

**Published:** 2022-01-04

**Authors:** Zihao Fan, Yuxian Li, Sisi Chen, Ling Xu, Yuan Tian, Yaling Cao, Zhenzhen Pan, Xiangying Zhang, Yu Chen, Feng Ren

**Affiliations:** Beijing Youan Hospital, Capital Medical University, Beijing, China

**Keywords:** magnesium isoglycyrrhizinate, concanavalin A, autophagy, liver injury, autophagic cell death

## Abstract

**Background and Aims:** Acute liver failure (ALF) is a type of liver injury that is caused by multiple factors and leads to severe liver dysfunction; however, current treatments for ALF are insufficient. Magnesium isoglycyrrhizinate (MgIG), a novel glycyrrhizin extracted from the traditional Chinese medicine licorice, has a significant protective effect against concanavalin A (ConA)-induced liver injury, but its underlying therapeutic mechanism is unclear. Hence, this study aims to explore the potential therapeutic mechanism of MgIG against ConA-induced immune liver injury.

**Methods:** ConA (20 mg/kg, i. v.) was administered for 12 h to construct an immune liver injury model, and the treatment group was given MgIG (30 mg/kg, i. p.) injection 1 h in advance. Lethality, liver injury, cytokine levels, and hepatocyte death were evaluated. The level of autophagy was evaluated by electron microscopy, RT-PCR and western blotting, and hepatocyte death was assessed *in vitro* by flow cytometry.

**Results:** MgIG significantly increased the survival rate of mice and ameliorated severe liver injury mediated by ConA. The decrease in the number of autophagosomes, downregulation of LC3b expression and upregulation of p62 expression indicated that MgIG significantly inhibited ConA-induced autophagy in the liver. Reactivation of autophagy by rapamycin (RAPA) reversed the protective effect of MgIG against ConA-induced liver injury. Compared with MgIG treatment, activation of autophagy by RAPA also promoted the expression of liver inflammation markers (IL-1β, IL-6, TNF-α, CXCL-1, CXCL-2, CXCL-10, etc.) and hepatocyte death. *In vitro* experiments also showed that MgIG reduced ConA-induced hepatocyte death but did not decrease hepatocyte apoptosis by inhibiting autophagy.

**Conclusion:** MgIG significantly ameliorated ConA-induced immune liver injury in mice by inhibiting autophagy. This study provides theoretical support for the ability of MgIG to protect against liver injury in clinical practice.

## Introduction

Acute liver failure (ALF) is a severe form of liver dysfunction characterized by abnormal liver biochemical indicators, jaundice, and coagulation dysfunction as the main clinical manifestations, and approximately half of the patients experience multiple organ failure and death; thus, ALF imposes a heavy burden on society (Wendon et al., 2017; Weiler et al., 2020). Although the use of liver transplantation has drastically improved the survival rate, there are still many deficiencies in the treatment of ALF (Olivo et al., 2018).

**FIGURE 1:**
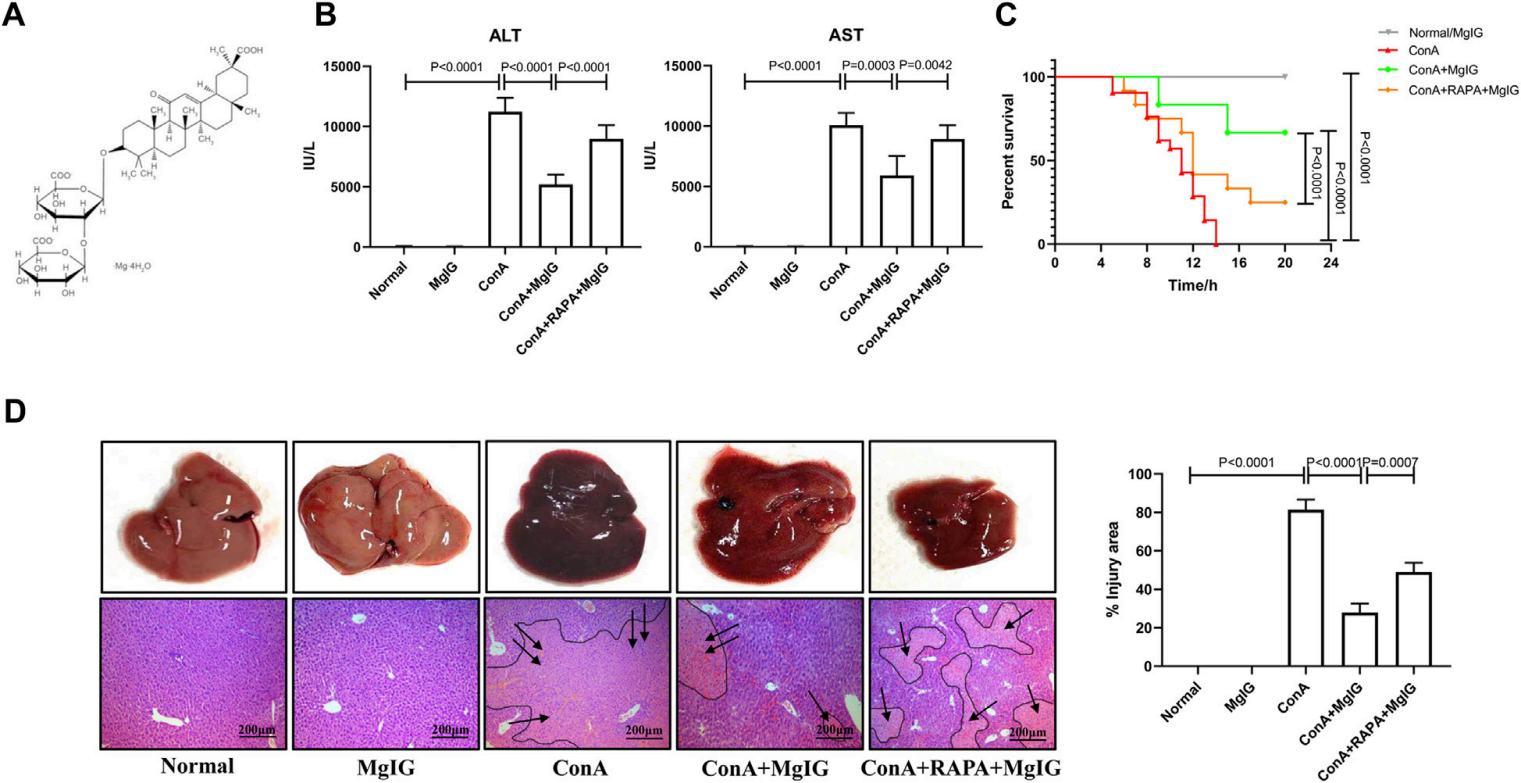
Pretreatment with MgIG ameliorates ConA-induced liver injury in mice. Liver injury was induced by ConA (20 mg/kg, iv, n = 8) in mice in the model group. MgIG-treated group mice were administered MgIG (30 mg/kg, ip) 1 h prior to ConA treatment (n = 8). Mice in the RAPA intervention group were administered RAPA (2 mg/kg, ip) 1 h prior to ConA treatment (n = 8). Control mice were pretreated with saline/MgIG 1 h prior to ConA administration (n = 4). The data are shown as the mean and SD of at least three independent experiments. **(A)** Chemical structure of MgIG. **(B)** Serum AST and ALT levels in the different groups. **(C)** Survival curves of the different groups. The administration of MgIG significantly reduced the mortality of ConA-treated mice. **(D)** Representative images of liver tissues and H&E-stained liver sections. The areas surrounded by a black line and arrow are areas of liver tissue damage (magnification: 200×).

Licorice is a common herbal medicine that has been used in traditional Chinese medicine for centuries. Studies have shown that a variety of extracts have many pharmacological activities, such as antiviral, antimicrobial, anti-inflammatory, antitumor and other activities (Richard, 2021). Magnesium isoglycyrrhizinate (MgIG) is a magnesium salt mainly composed of 18α-glycyrrhizic acid stereoisomers (Figure 1A) and belongs to the fourth generation of glycyrrhizic acid preparations. As a novel glycyrrhizic acid extracted from the traditional Chinese medicine licorice, MgIG has anti-inflammatory, antioxidant, antiviral, immunoregulatory and hepatocellular protective effects (Tang et al., 2021). MgIG can protect the liver by regulating lipid metabolism in a mouse model of nonalcoholic fatty liver disease (Jiang et al., 2020), and MgIG can inhibit hepatotoxicity by inhibiting oxidative stress, inflammation, and apoptosis in a mouse model of arsenic trioxide induced acute liver injury (Liu et al., 2021). Therefore, MgIG is widely used as a hepatoprotective agent to ameliorate liver injury and improve liver function. However, there has been little research on the mechanism underlying the effect of MgIG against concanavalin A (ConA)-induced immune liver injury.

ConA, a lectin-like polysaccharide extracted from plants, has been successfully applied to establish an immune hepatitis model in mice and is currently used to simulate hepatitis B-related immune liver injury (Tiegs et al., 1992; Gao et al., 2019). Autophagy, a physiological process in which cells degrade harmful components and recycle them to achieve the needs of cell metabolism and the renewal of some organelles, plays different roles in various models of severe hepatitis-related liver failure. In D-GalN/LPS induced liver injury in mice, an increase in the level of autophagy may play a protective role in the liver. In contrast, autophagy tends to promote injury in ConA-induced liver injury in mice (Tian et al., 2021). Studies have shown that ConA induces dendritic cell activation by enhancing autophagy, which exacerbates autoimmune hepatitis (Fan et al., 2020). Furthermore, it is worth noting that ConA can mediate hepatocyte death through mitochondrial autophagy; thus, ConA is the focus of many antitumor studies, especially those involving the inhibition of hepatoma cells (Chang et al., 2007; Lai et al., 2015). Thus, abnormal autophagic flux may be detrimental either via its pro-survival effects (such as in cancer progression) or its possible cell death-promoting effects (Levine and Kroemer, 2019). Inhibition of aberrant autophagic flux may be a new therapeutic approach for ConA-induced immune liver injury.

Previously, we demonstrated that MgIG exerts a significant protective effect against ConA-induced liver injury by reducing inflammation (Gao et al., 2020a), but its curative mechanism is not very clear. This study aims to explore the potential therapeutic mechanism of MgIG against ConA-induced immune liver injury and, for the first time, reveals that MgIG can ameliorate ConA-induced liver injury by inhibiting autophagy.

## Materials and Methods

### Animals and Treatment

All experiments were performed strictly in accordance with the ethical guidelines of the Capital Medical University Animal Experimentation Committee. After the animal protocol was approved by the Institutional Animal Care and Use Committee (IACUC) of Capital Medical University (CMU, Beijing, China), male wild-type (WT; Balb/c) mice (8–10 weeks old) were purchased from Weitong Lihua Company (Hebei, China). During the study, all animals were kept in CMU animal facility with pathogen-free environmental conditions with an air‐conditioned room at 23 ± 2°C with a 12‐h light/dark cycle. Additionally, animals were provided with water and food ad libitum and allowed to acclimate to the conditions of the animal center for a week prior to the start of experiments.

A total of 32 mice were randomly assigned to five groups: the normal control group (n = 4), MgIG control group (n = 4), ConA model group (n = 8), MgIG treatment group (n = 8), and rapamycin (RAPA) intervention group (n = 8). Mice in the ConA model group, MgIG treatment group and RAPA intervention group were injected via the tail vein with ConA (20 mg/kg; Sigma, United States) dissolved in phosphate-buffered saline (PBS) to induce ALF. The normal control group and MgIG control group were injected with an equal volume of PBS according to the body weight of the mice. MgIG (30 mg/kg; Chia-tai Tianqing Pharmaceutical Co., Ltd., China) dissolved in 0.9% physiological saline was administered intraperitoneally to mice in the MgIG control group, MgIG treatment group, and RAPA intervention group 1 h prior to ConA administration. The autophagy activator RAPA (2 mg/kg; Sigma, CHN) was injected intraperitoneally into mice in the RAPA intervention group 1 h prior to ConA administration. Twelve hours after ConA treatment, the mice were sacrificed after injection of an anesthetic (4% chloral hydrate, 0.20 ml/20 g), and liver tissues and peripheral blood serum were collected. Serum samples and liver tissue samples were stored at -80 °C for later use.

### Liver Injury Assessment

First, as markers of liver injury, the levels of serum alanine aminotransferase (ALT) and aspartate aminotransferase (AST) were measured using a multiparameter analyzer (AU 5400, Olympus, Japan). Then, pathological sections were observed. After liver tissues fixed in formalin were embedded in paraffin, they were stained with hematoxylin and eosin (H&E) according to a standard method and then analyzed with an optical microscope.

### Electron Microscopy

Autophagosomes were observed, and the number of autophagosomes in liver samples from mice was determined by transmission electron microscopy. The analysis was performed as described previously (Zhang et al., 2017).

### Reverse Transcription-Quantitative Polymerase Chain Reaction Analysis

The liver tissues of mice were homogenized and ground in TRIzol reagent (Invitrogen Life Technologies, Carlsbad, CA) to extract total RNA. Then, the SuperScriptTM III First-Strand Synthesis System (Invitrogen, Carlsbad, CA, USA) was used to reverse-transcribe the extracted RNA into cDNA. The volume of each PCR system was 20 μl, which included 4 μl cDNA, 0.4 μl forward and reverse primers, 5.2 μl enzyme-free sterile water and 10 μl SYBR Green (Platinum SYBR Green qPCR kit, Invitrogen). The PCR conditions were as follows: 50°C for 2 min and 95°C for 5 min followed by 44 cycles of 95°C for 15 s, 60°C for 30 s, and 55°C for 4 s. The levels of target mRNAs were analyzed by the 2−ΔΔCt method and standardized to the mRNA level of HPRT.

### Western Blot Analysis

Total protein was extracted from primary hepatocytes or mouse liver tissue using RIPA lysis working solution containing protease and phosphatase inhibitors. Protein quantification was carried out using a bicinchoninic acid (BCA) protein determination kit (Biomed, Beijing, China) according to the manufacturer’s instructions. The proteins were first separated by SDS-12% polyacrylamide gel electrophoresis and then transferred to a PVDF membrane overnight at 4°C. *β*-Actin, LC3b, p62, caspase-3, cleaved caspase-3, Bax, and Bcl-2 primary antibodies (1:1000, Cell Signaling Technology, Danvers, MA, United States) were diluted in 5% nonfat milk in TBST, and then the membrane was incubated with the antibodies at 4°C overnight with slow shaking. After being washed in TBST for 90 min, the membrane was incubated with horseradish peroxidase-conjugated secondary antibody (1:2000, Cell Signaling Technology) for 60 min at room temperature and then washed in TBST for 90 min. An enhanced chemiluminescence kit (Thermo Fisher Scientific, Rockford, IL) was used for exposure and development of the target protein bands.

### Immunofluorescence

Paraffin sections of the liver were deparaffinized and fixed with cold methanol for 10 min and then permeabilized with 0.1% Triton X-100 in PBS. After the sections were blocked with 1% goat serum at 37°C for 1 h, they were incubated with a rabbit LC3 antibody (1:500, Cell Signaling Technology) at 4 °C overnight. The next day, after being washed with PBS, the sections were incubated with an Alexa Fluor 488-conjugated goat anti-rabbit IgG antibody (1:250, Invitrogen, Thermo Fisher Scientific, Inc.) at 37°C for 30 min. After the sections were washed again with PBS, they were stained with 6-diamino-2-phenylindole (DAPI, 1 μg/ml, Abcam) for 10 min and observed under a Leica DM2500 fluorescence microscope.

### Serum Cytokine Level Measurement

Serum cytokine levels were measured using the Luminex Milliplex^®^ MAP Kit (Mouse Cytokine/Chemokine Magnetic Bead Panel, 96-Well Plate Assay, Merck, United States) according to the manufacturer’s instructions, and the results were analyzed using a Luminex 200 system.

### Terminal Deoxynucleotidyl Transferase dUTP Nick-End Labeling Assay

An *in-situ* cell death detection kit (KeyGen BioTECH) was used to detect apoptotic cells in liver slices, is mainly through FITC-streptomycin-biotin labeling (TUNEL staining, green fluorescence). Negative control slices not subjected to TUNEL staining and positive control slices treated with DNase were analyzed. The slices were stained with DAPI (1 μg/ml, Abcam) for 10 min and observed under a Leica DM2500 fluorescence microscope.

### Caspase-3 Activity Assay

Caspase-3 activity in mouse liver tissue was measured using a Caspase-3 activity kit (Beyotime Shanghai, China) according to the manufacturer’s instructions.

### Cell Culture and Treatments

According to a previously described method (Charni-Natan and Goldstein, 2020), primary hepatocytes were extracted from Balb/c mice. The extracted mouse primary hepatocytes were spread on a collagen-coated cell plate and incubated in Dulbecco’s modified Eagle’s medium (DMEM, Thermo Fisher, Inc., Rockford, IL, United States) supplemented with 10% fetal bovine serum (FBS, Thermo Fisher, Inc.) and 1% pen-strep (PS, Thermo Fisher, Inc.) at 37°C and 5% CO2. To determine the optimal exposure time and concentration, primary hepatocytes were stimulated with ConA (20 μg/ml) for different times (0, 3, 6, 12, 24, or 48 h) or stimulated with ConA at different concentrations (0, 1, 5, 10, or 20 μg/ml) for 24 h. Additionally, primary hepatocytes were incubated with different concentrations of MgIG (5, 20, 80, or 320 μg/ml) for 1 h before being treated with ConA (20 μg/ml) for 24 h. In the experiment using the autophagy activator RAPA, primary hepatocytes were incubated with MgIG (80 μg/ml) and RAPA (10 μg/ml) for 1 h and then treated with ConA (20 μg/ml) for 24 h.

### Flow Cytometry

SYTOX^®^ Green Nucleic Acid Stain (1 μM, Invitrogen, Inc.) was used to detect cell death. Mouse primary hepatocytes were seeded in 6-well plates at a density of 1 × 10^6^ cells/well. After exposure to drugs, the cells were resuspended in PBS and then stained with SYTOX reagent in the dark for 15 min. A FACScan flow cytometer (BD Bioscience) was used to analyze the samples, and the data were analyzed with FlowJo software.

### Statistical Analysis

All experiments were conducted at least 3 times independently, and the experimental results are expressed as the average and standard deviation. Unpaired *t*-test, one-way analysis of variance (ANOVA) with Dunnett’s or Tukey’s were used for statistical analysis to calculate *p* values, and *p* values less than 0.05 were considered statistically significant.

## Results

### MgIG Ameliorates ConA-Induced Liver Injury

The results showed that MgIG significantly ameliorated acute liver injury in mice. Compared with those in the ConA model group, the serum ALT and AST levels in the MgIG treatment group were significantly reduced (Figure 1B). Moreover, survival analysis showed that MgIG treatment significantly prolonged the survival time of mice (Figure 1C). Compared with that of mice in the ConA model group, the overall liver morphology of the mice in the MgIG treatment group was significantly improved, and liver congestion was reduced. H&E staining of liver tissue sections showed that the liver tissue in the ConA model group was severely congested and showed large necrotic areas, while liver injury in the MgIG treatment group was significantly reduced (Figure 1D). Therefore, MgIG pretreatment significantly ameliorates ConA-induced liver damage and improves the survival rate of mice.

### MgIG Inhibits ConA-Induced Liver Autophagy

**FIGURE 2:**
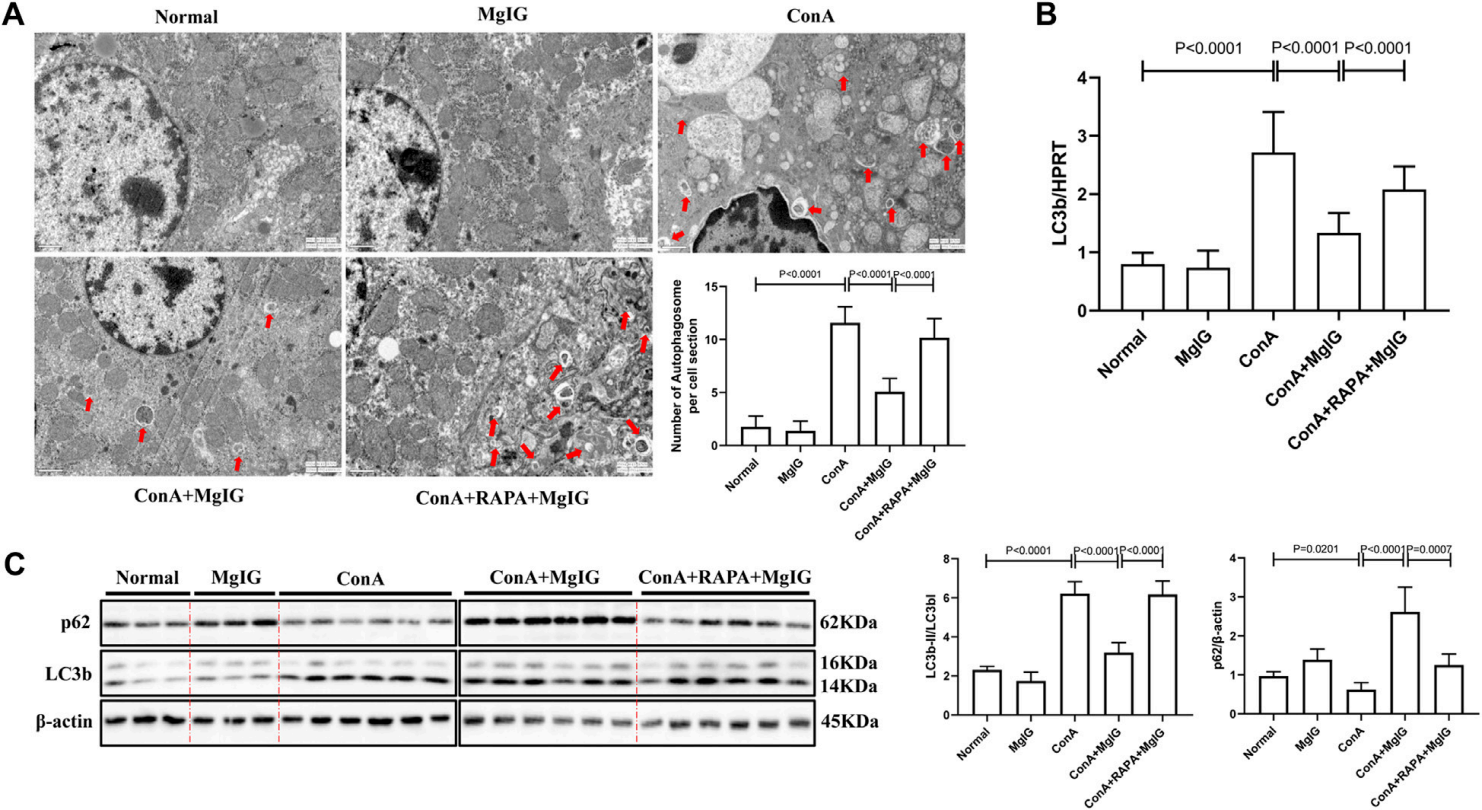
MgIG inhibits liver autophagy induced by ConA. The data are shown as the mean and SD of at least three independent experiments. **(A)** Representative electron microscopy image of autophagosomes in the mouse liver. The arrows indicate autophagosomes (magnification: 15000×). **(B)** qRT-PCR was used to measure the expression level of the LC3b gene in mouse liver tissue, with the housekeeping gene HPRT as a control. **(C)** The expression levels of autophagy-related proteins, including LC3 and p62, in mouse liver tissues were measured by western blotting, with *β*-actin as a control.

Electron microscopy showed that the number of autophagosomes was increased in ConA-induced liver injury. Compared with that in the ConA model group, the number of autophagosomes in the MgIG treatment group was significantly reduced (Figure 2A). The western blot and PCR results (Figures 2B,C) also showed that autophagy was activated, the conversion of LC3-I to LC3-II increased, and p62 expression decreased in the ConA model group. In the MgIG treatment group, autophagy was inhibited, the conversion of LC3-I to LC3-II was reduced, and the accumulation of p62 was increased. Immunofluorescence experiments confirmed these results. According to LC3 fluorescence staining, the number of LC3 fluorescence particles in the MgIG treatment group was decreased (Supplementary Figure S1). These results show that MgIG inhibits autophagy in liver tissue in ConA-induced liver injury.

### MgIG Ameliorates ConA-Induced Liver Injury by Inhibiting Autophagy

The activation of autophagy by RAPA reversed the protective effect of MgIG. The number of autophagosomes in liver tissues in the RAPA intervention group was significantly increased compared with that in the MgIG treatment group (Figure 2A). Moreover, LC3 expression was increased and p62 expression was decreased (Figures 2B,C). The serum levels of ALT and AST were increased, the survival rate was decreased, and the area of liver tissue necrosis was expanded in the RAPA intervention group compared with the MgIG treatment group (Figures 1B–D). Thus, MgIG ameliorates ConA-induced liver injury by inhibiting autophagy.

### MgIG Regulates Inflammatory Cytokine Levels by Inhibiting Autophagy

**FIGURE 3:**
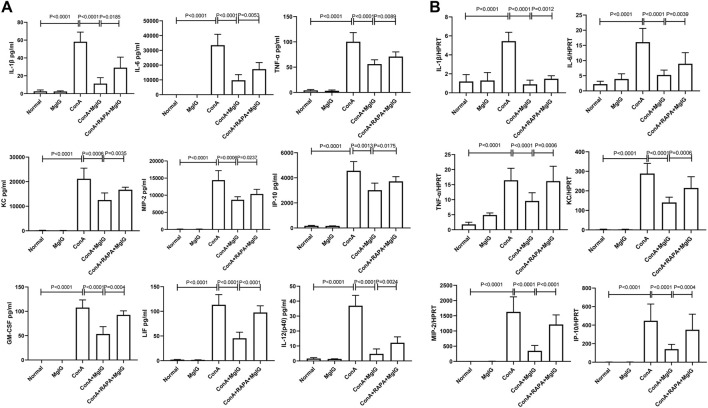
MgIG regulates cytokine levels in the serum and cytokine transcript levels in liver tissues in ConA-induced liver injury. The data are shown as the mean and SD of at least three independent experiments. **(A)** Measurement of cytokine levels in the sera of mice in each model group by the Luminex Milliplex^®^ MAP Kit (IL-1β, IL-6, TNF-α, KC, MIP-2, IP-10, GM-CSF, LIF, IL-12). **(B)** Analysis of the gene expression levels of cytokines (IL-1β, IL-6, TNF-α, KC, MIP-2, IP-10) in liver tissues from each group by qRT-PCR.

The previous studies have shown that MgIG has anti-inflammatory effects (Gao et al., 2020a). Furthermore, in mouse bone marrow-derived macrophages (BMMs) in which the inflammatory response was induced by LPS, MgIG effectively inhibited the expression of inflammatory factor genes and inflammation-related proteins (Supplementary Figure S2, 3). Therefore, we further investigated whether MgIG regulates inflammation by inhibiting autophagy. Analysis of cytokine levels in the sera of mice showed that the expression of some inflammation-related cytokines, such as IL-1β, IL-6, TNF-α, KC, MIP-2, IP-10, GM-CSF, LIF, and IL- 12 (p40), was significantly increased in the ConA model group and decreased in the MgIG treatment group. RAPA-induced activation of autophagy successfully reversed the protective effect of MgIG. IL-1β, IL-6, TNF-α, KC, MIP-2, IP-10, GM-CSF, LIF, IL-12 (p40) expression was increased in the RAPA intervention group compared with the MgIG treatment group (Figure 3A). The results of mRNA expression of corresponding inflammation-related cytokines in liver tissues showed that the trend in the expression of cytokines such as IL-1β, IL-6, TNF-α, KC, MIP-2, and IP-10 was consistent with that in peripheral blood (Figure 3B). However, there was no significant difference in the expression of GM-CSF, LIF, or IL-12 (p40) in the liver tissues between any groups. Moreover, we measured the levels of 20 cytokines, such as IL-1α, in the peripheral blood of mice, and the results showed that the expression of some cytokines was not regulated by autophagy or was not significantly regulated (Supplementary Figure S4). The above results indicate that MgIG regulates inflammatory factors by inhibiting autophagy to play a protective role but that this is not the main pathway underlying the effect of MgIG.

### MgIG Reduces Hepatocyte Death in Tissue Affected by ConA-Induced Liver Injury

**FIGURE 4:**
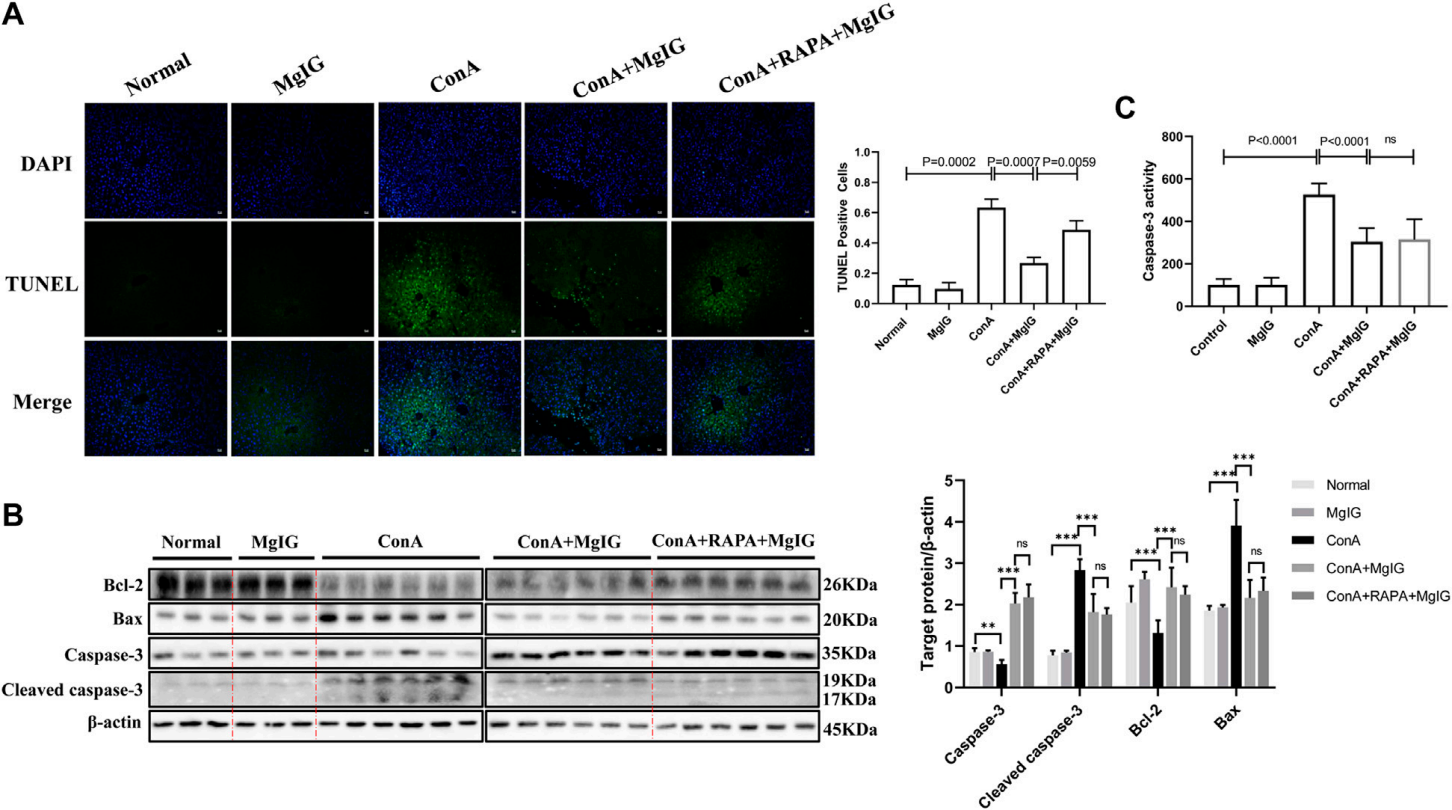
MgIG reduces hepatocyte death in liver tissues affected by ConA-induced liver injury. The data are shown as the mean and SD of at least three independent experiments. **(A)** Representative images of TUNEL-stained frozen liver tissue sections at 100× magnification. Quantification of stained cells to evaluate hepatocyte death in liver tissue. **(B)** Caspase-3, cleaved caspase-3, Bax and Bcl-2 expression levels in liver tissue were measured by western blotting, with *β*-actin as a control (^***^ *p* < 0.001, ^**^ *p* < 0.01). **(C)** Analysis of caspase-3 activity levels in liver tissue.

The results of TUNEL staining of showed that the number of apoptotic hepatocytes was significantly increased in ConA-induced liver injury and that MgIG pretreatment significantly decreased hepatocyte apoptosis in liver tissue (Figure 4A). This finding was supported by the level of the apoptosis-related protein caspase-3, cleaved caspase-3, Bax and Bcl-2 in the mouse liver determined by western blotting (Figure 4B). In addition, MgIG pretreatment significantly reduced the activity of caspase-3 (Figure 4C). When RAPA was used to activate autophagy, TUNEL staining showed that apoptosis increased again, as expected (Figure 4A). However, there was no significant difference in caspase-3, cleaved caspase-3, Bax and Bcl-2 protein expression, and caspase-3 activity between the RAPA intervention group and the MgIG treatment group (Figures 4B,C). This result confirms that MgIG reduces hepatocyte death in tissue affected by ConA-induced liver injury.

### MgIG Does Inhibit Autophagy to Reduce Hepatocyte Death but Does Not Decrease Hepatocyte Apoptosis *in vitro*

**FIGURE 5:**
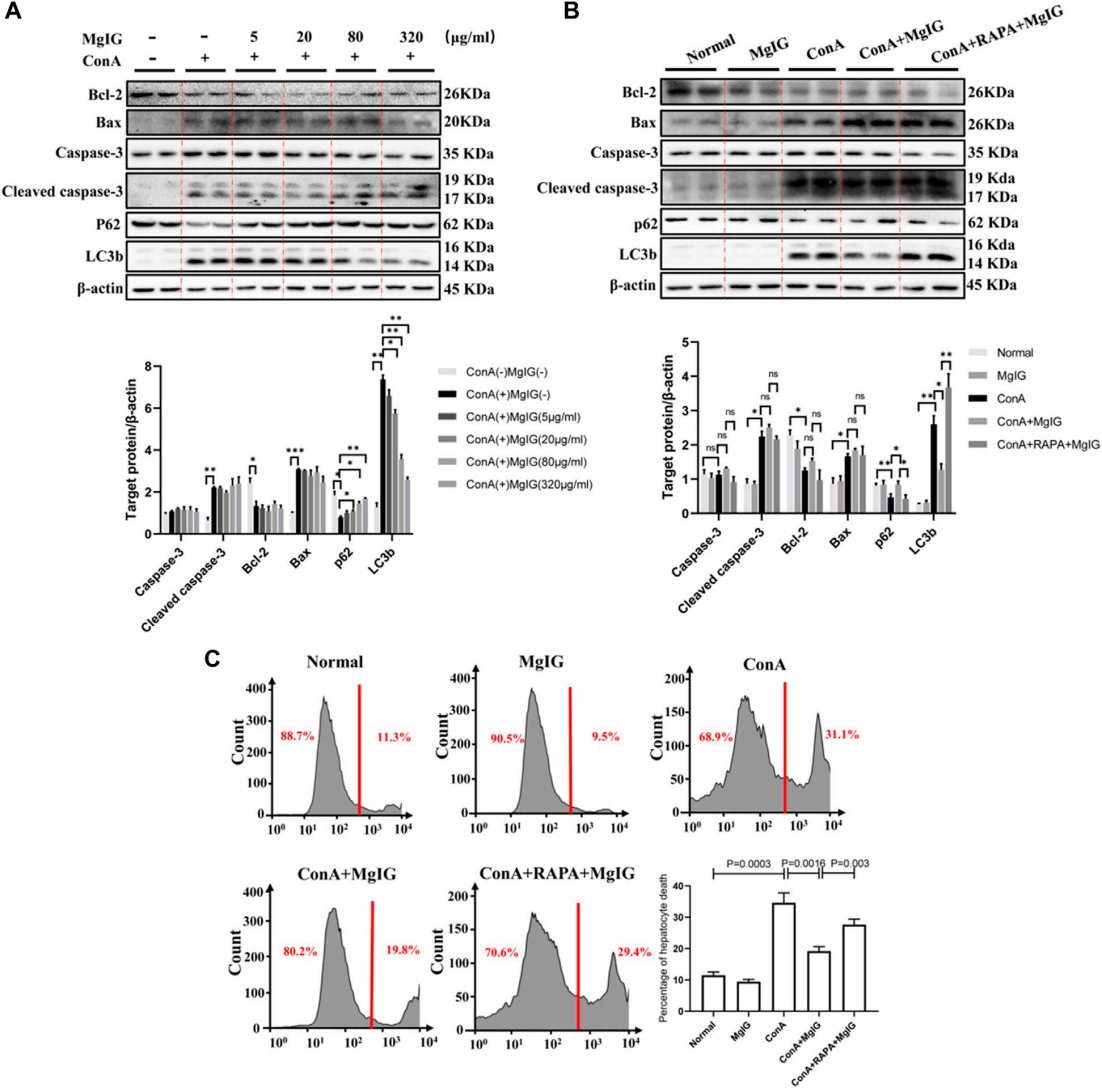
MgIG reduces hepatocyte death by inhibiting autophagy *in vitro*. The data are shown as the mean and SD of at least three independent experiments. **(A)** The effects of different concentrations of MgIG (5, 20, 80, 320 μg/ml) on protein (caspase-3, cleaved caspase-3, Bax, Bcl-2, P62 and LC3b) expression levels *in vitro* were determined by western blotting, with *β*-actin as a control ( ^***^ *p* < 0.001, ^**^ *p* < 0.01, ^*^ *p* < 0.05). **(B)** The protein expression levels of caspase-3, cleaved caspase-3, Bax, Bcl-2, p62 and LC3b in the normal control, MgIG (80 μg/ml) control, ConA (20 μg/ml) model, MgIG (80 μg/ml) treatment and RAPA (10 μg/ml) intervention groups were determined by western blotting, with *β*-actin as a control (^**^ *p* < 0.01, ^*^ *p* < 0.05). **(C)** Hepatocytes were stained with SYTOX^®^ Green Nucleic Acid staining reagent *in vitro*, and hepatocyte mortality was measured and quantified by flow cytometry.

First and foremost, mouse primary hepatocytes were treated with ConA at different concentrations or different times, and the LC3 level increased continuously, indicating that ConA successfully induced autophagy in mouse primary hepatocytes (Supplementary Figure S5). MgIG at different concentrations treatment had no cytotoxicity effect on mouse primary hepatocytes, and MgIG significantly decreased hepatocyte cytotoxicity after ConA exposure (Supplementary Figure S6). Furthermore, hepatocytes were pretreated with various concentrations of MgIG 1 hour before ConA administration, and the results showed that as the MgIG concentration gradually increased, autophagy was gradually inhibited, as manifested by a gradual decrease in the level of LC3 and a gradual increase in the level of p62. However, the difference in caspase-3, cleaved caspase-3, Bax and Bcl-2 levels was not obvious after MgIG treatment (Figure 5A). When the autophagy activator RAPA was administered, the change in autophagy level was successfully reversed, the LC3 level increased and the p62 level decreased. The level of the apoptosis-related protein caspase-3, cleaved caspase-3, Bax and Bcl-2 was no significant difference (Figure 5B). Importantly, we determined the number of dead hepatocytes using flow cytometry. The number of dead hepatocytes was significantly reduced in the MgIG treatment group compared with the ConA model group, but RAPA intervention reversed the reduction in hepatocyte death (Figure 5C). Moreover, we measured the number of hepatocytes stained by apoptotic reagents using flow cytometry, and the results showed that there was no significant difference in the level of apoptosis between any of the groups (Supplementary Figure S7). The above results show that MgIG reduces hepatocyte death but does not decrease hepatocyte apoptosis by inhibiting autophagy *in vitro*.

## Discussion

**FIGURE 6:**
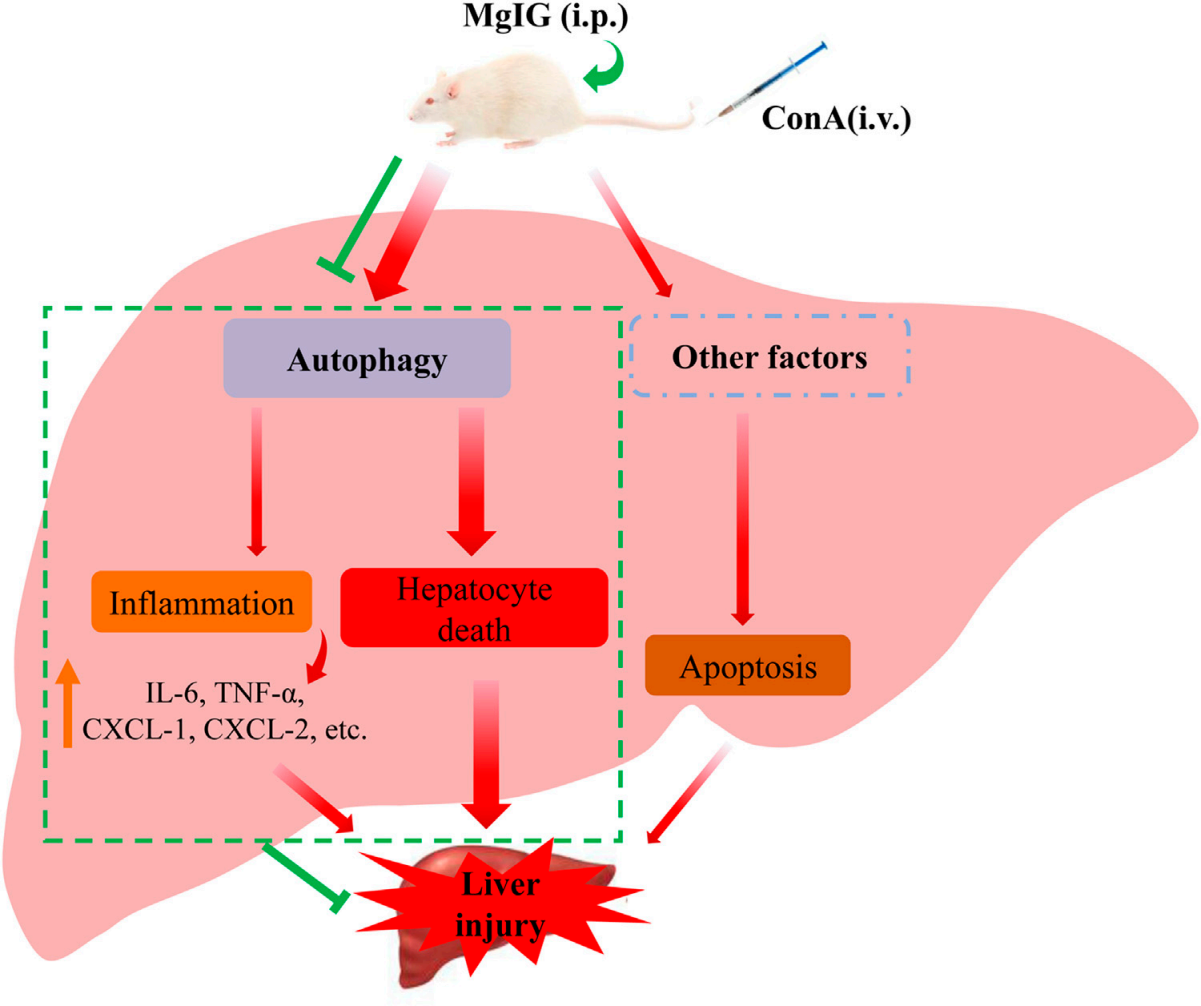
Proposed mechanism underlying the positive effects of MgIG on ConA-induced liver injury in mice. MgIG inhibits autophagy, thereby reducing the direct damage caused by abnormal autophagic flux, which is the primary molecular mechanism. Furthermore, MgIG inhibits the expression of some inflammatory factors by inhibiting autophagy to attenuate the inflammatory response, thereby indirectly reducing liver damage.

Our study clarified that MgIG played a significant role in ameliorating ConA-induced liver injury in mice and then proved that MgIG can ameliorate liver injury by inhibiting autophagy. MgIG inhibits the expression of some inflammatory factors by inhibiting autophagy to attenuate the inflammatory response, thereby indirectly ameliorating liver damage. In addition, studies have shown that MgIG alleviates the direct damage caused by abnormal autophagy flux by inhibiting autophagy, mainly by reducing the death of hepatocytes rather than inhibiting apoptosis. (Figure 6).

Autophagy is a vital physiological process that depends on self-catabolism to achieve the turnover of intracellular substances and promotes homeostasis by removing misfolded proteins and damaged organelles during this process (Mizushima et al., 2008; Kim and Lee, 2014). Autophagy is inextricably linked with hepatocyte metabolism and the occurrence and development of liver diseases. It not only regulates hepatocyte function but also affects nonparenchymal cells such as macrophages and endothelial and hepatic stellate cells. Additionally, the abnormal regulation of autophagy is closely related to many liver diseases (Allaire et al., 2019; Gao et al., 2020b). Studies have shown that the loss of autophagy can promote spontaneous hepatomegaly and liver injury in an Atg5-shRNA mouse model but that liver damage can be significantly alleviated after intervention with autophagy activators (Cassidy et al., 2018). Moreover, autophagy plays a key role in restricting various types of stress-related liver injury and even hepatocyte death caused by hepatotoxic drugs, ischemia-reperfusion and so on (Xue et al., 2020; Zhang et al., 2020). However, every coin has two sides: autophagy can act as a temporary protective mechanism during transient stress but can also promote cell death, especially in the case of prolonged autophagy or excessive autophagy. A robust autophagy response is usually harmful, especially when the circulation of cellular components in the cell pool is disrupted (Brunk and Terman, 2002; Pattingre et al., 2005), or enhances harmful side effects such as the formation of pathogenic *β*-amyloid (Yu et al., 2005). Studies have found that promoting autophagy seems to make neurons and glial cells more likely to die (Eskelinen et al., 2003; Shacka et al., 2006). Regulating autophagy has emerged as a potential new therapeutic strategy when autophagy itself becomes a risk factor. In our study, the main mechanism of ConA-induced liver injury in mice was increased autophagic flux. MgIG significantly reduced liver injury while also inhibiting autophagy, and the protective effect of MgIG was reversed by inhibiting the kinase mTOR to activate autophagy.

We have demonstrated that MgIG alleviates liver damage by decreasing liver inflammation through regulating the p38 and JNK MAPK signaling pathways (Gao et al., 2020a), and this study further verified the that MgIG exerts an anti-inflammatory effect by regulating the autophagy pathway. In fact, the role of autophagy in inflammatory regulation has received increasing attention as the understanding of the mechanism of autophagy and inflammation has improved (Jin and Zhang, 2020). A recent study showed that autophagy promoted by the inactivation of mTOR is the main mechanism of injury in airway inflammation induced by particulate matter (Wu et al., 2020). In addition, researchers studied the function of autophagy and its related proteins in inflammation and concluded that autophagy-related proteins are involved in the functions of almost all cell types involved in inflammation and that inhibiting or augmenting autophagy has dramatic effects in a multitude of cell culture and animal models of inflammatory disorders (Matsuzawa-Ishimoto et al., 2018). Hence, increased attention has been paid to strategies that target autophagy to decrease the inflammatory response. In this study, MgIG reduced inflammation by inhibiting autophagy, which was mainly manifested by a reduction in the expression of inflammatory factors in the sera of mice and of the expression of corresponding inflammatory genes in liver tissue. When autophagy is activated, the effect of MgIG on reducing inflammation is reversed, which further explains the regulatory effect of autophagy on inflammation. However, in our analysis of the levels of various inflammation-related cytokines in the mouse serum, not all inflammation-related cytokines were affected by autophagy regulation. This may have been because the regulation of inflammation by autophagy is only part of the molecular mechanism underlying ConA-induced liver injury in mice. This further illustrates the exacerbating effect of autophagy itself on liver injury in mice.

MgIG mainly exerts its protective effect against ConA-induced autophagic cell death (ACD) by inhibiting autophagy. ConA can induce T cell mitogens in autoimmune hepatitis to cause immune damage (Kaneko et al., 2000); moreover, it exhibits a certain level of cytotoxicity: ConA is internalized into the cell after binding to the mannose residues of polysaccharides or glycoproteins on the cell membrane and then preferentially aggregates on mitochondria to cause changes in the permeability of the mitochondrial membrane. Although it is possible that ConA induces apoptosis, this was not the main molecular mechanism. Cell apoptosis was detected by Annexin V-phycoerythrin (PE)/7-amino-actinomycin (7-AAD) double staining, there was little difference between the groups. Conversely, the level of autophagy increases after hepatocytes exposed to ConA, leading to lysosomal degradation of the affected mitochondria and cell death (Lei and Chang, 2007). We used SYTOX Green Nucleic Acid Stain to stain dead cells (Roth et al., 1997)and then performed flow cytometric analysis, and we found that cells exposed to ConA exhibited autophagy-dependent death. It’s worth noting the role MgIG plays in this process. MgIG reduced cell death and inhibited autophagy under ConA exposure and activation of autophagy reversed this result. However, MgIG does not affect apoptosis *in vitro*. Meanwhile, we analyzed apoptosis *in vivo* mouse liver tissues. The results showed that cell apoptosis was significantly enhanced after ConA treatment and that the expression of apoptosis-related proteins was significantly changed in the ConA group compared with the control group. MgIG also affected tissue apoptosis and had a significant inhibitory effect. However, the activation of autophagy did not change this outcome. The activity and expression level of the apoptosis-related protein Caspase-3 showed no difference with the level of apoptosis in mouse liver tissues, but this does not mean that cells did not die. This further demonstrates the role of abnormal autophagy flux in promoting injury, and indicates that MgIG reduces liver injury by inhibiting autophagy. In fact, some studies have proposed that ACD, which is due to the accumulation of autophagosomes in dying cells, occurs, especially when autophagy is the key mechanism determining cell fate (Amelio et al., 2011). Research has also shown that Atg gene products are necessary for cell death (Bursch, 2001). Although autophagy is prominent in non-apoptotic forms of cell death, changes in necrosis, apoptosis and autophagy often occur simultaneously, especially *in vivo* (Clarke et al., 1998; Lemasters et al., 2002). This also explains the inconsistency in MgIG anti-apoptotic levels between the *in vivo* experiments and *in vitro* experiments.

As a new extract of Traditional Chinese medicine, the metabolic process of MgIG in the body is very complex. Whether metabolites of MgIG are more active in inhibition of autophagy and anti-inflammatory is worthy of further study. Moreover, the pathway still needs to make it further about MgIG inhibiting autophagy. In summary, we clarified the mechanism by which MgIG reduces hepatocyte death and the inflammatory response by inhibiting autophagy to ameliorate liver injury. This study provides proof that strategies that target autophagy can be used to treat diseases and a solid theoretical basis for the treatment of liver disease with MgIG.

## Data Availability

The original contributions presented in the study are included in the article/Supplementary Material, further inquiries can be directed to the corresponding authors.
