# Supplementary material for: Magnesium Isoglycyrrhizinate Ameliorates Concanavalin A-Induced Liver Injury by Inhibiting Autophagy
Source: Front Pharmacol. 2022 Jan 4;12:794319. doi: 10.3389/fphar.2021.794319 (PMC8763799; doi:10.3389/fphar.2021.794319)
Supplement: Supplementary file 1 [file DataSheet1.docx]

**Supplemental materials**

**
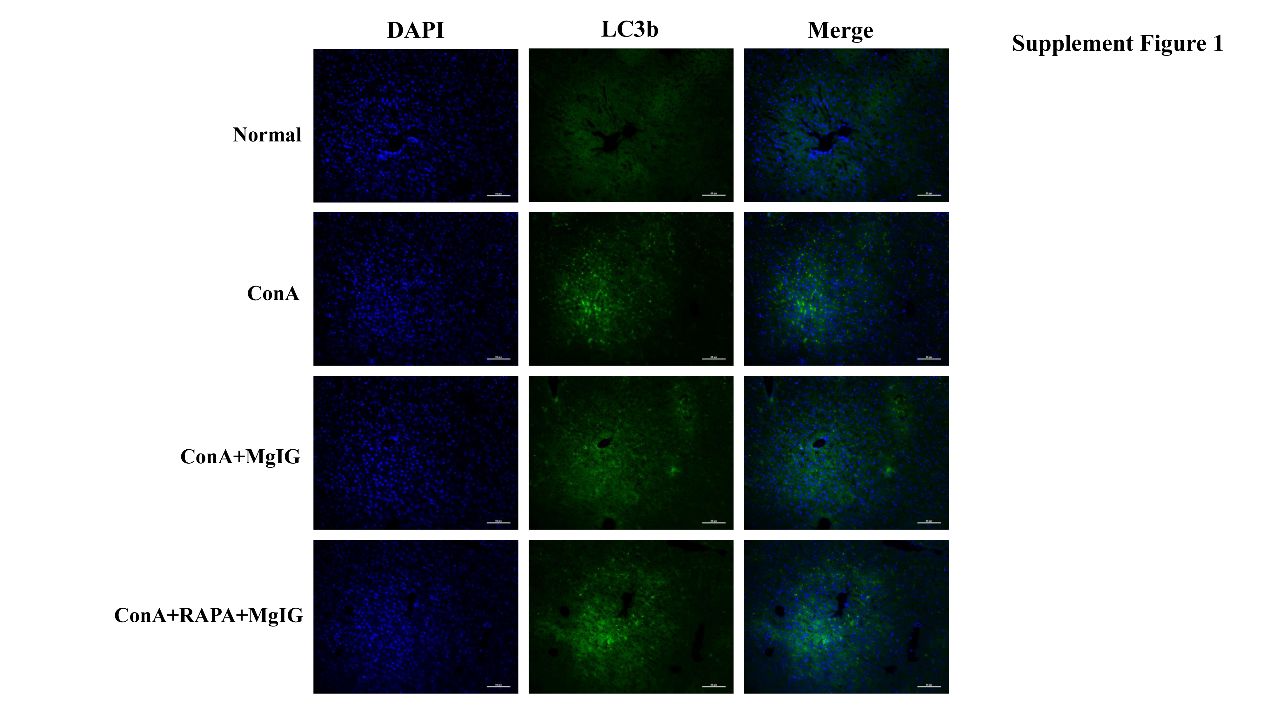
**

**S1. Immunofluorescence analysis of the expression of the autophagy-related protein LC3b in the liver tissues of mice.** At least three independent experiments were performed. Representative images of LC3b-stained frozen liver tissue sections at 200× magnification.


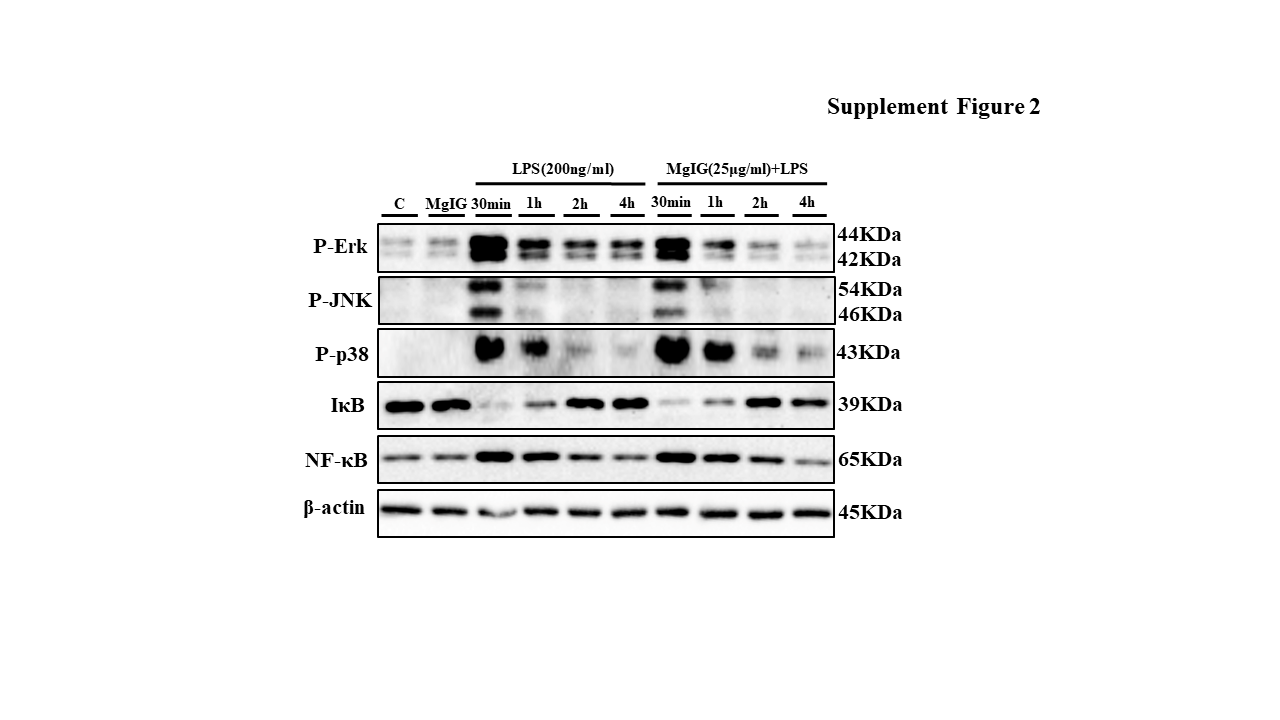


**S2. MgIG decreases the expression of LPS-induced** **inflammatory response-related proteins in** **murine BMMs.** At least three independent experiments were performed. BMMs were treated with LPS (200 ng/ml) for different times (30 minutes, 1 h, 2 h, or 4 h), and BMMs in the intervention group were pretreated with MgIG (25 μg/ml) for 1 h. The expression levels of inflammatory response-related proteins, including p-Erk, p-JNK, p-p38, IκB and NF-κB, in BMMs were measured by western blotting, with β-actin as a control.


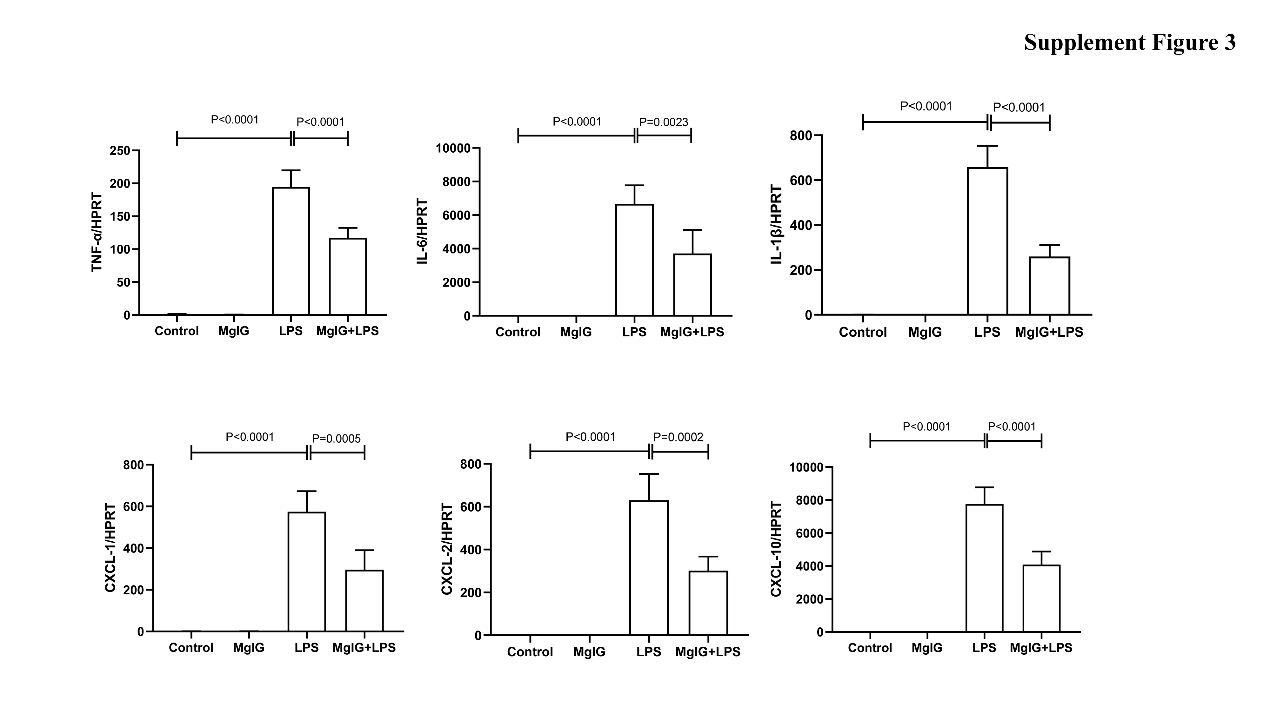


**S3. MgIG decreases the expression of LPS-induced inflammatory response-related genes in murine BMMs.** The data are shown as the mean and SD of at least three independent experiments. BMMs were stimulated with LPS (200 ng/ml) for 6 h, and BMMs in the intervention group were pretreated with MgIG (25 μg/ml) for 1 h. qRT-PCR was used to measure the expression levels of the IL-1β, IL-6, TNF-α, CXCL-1, CXCL-2, and CXCL-10 genes in BMMs, with the housekeeping gene HPRT as a control.


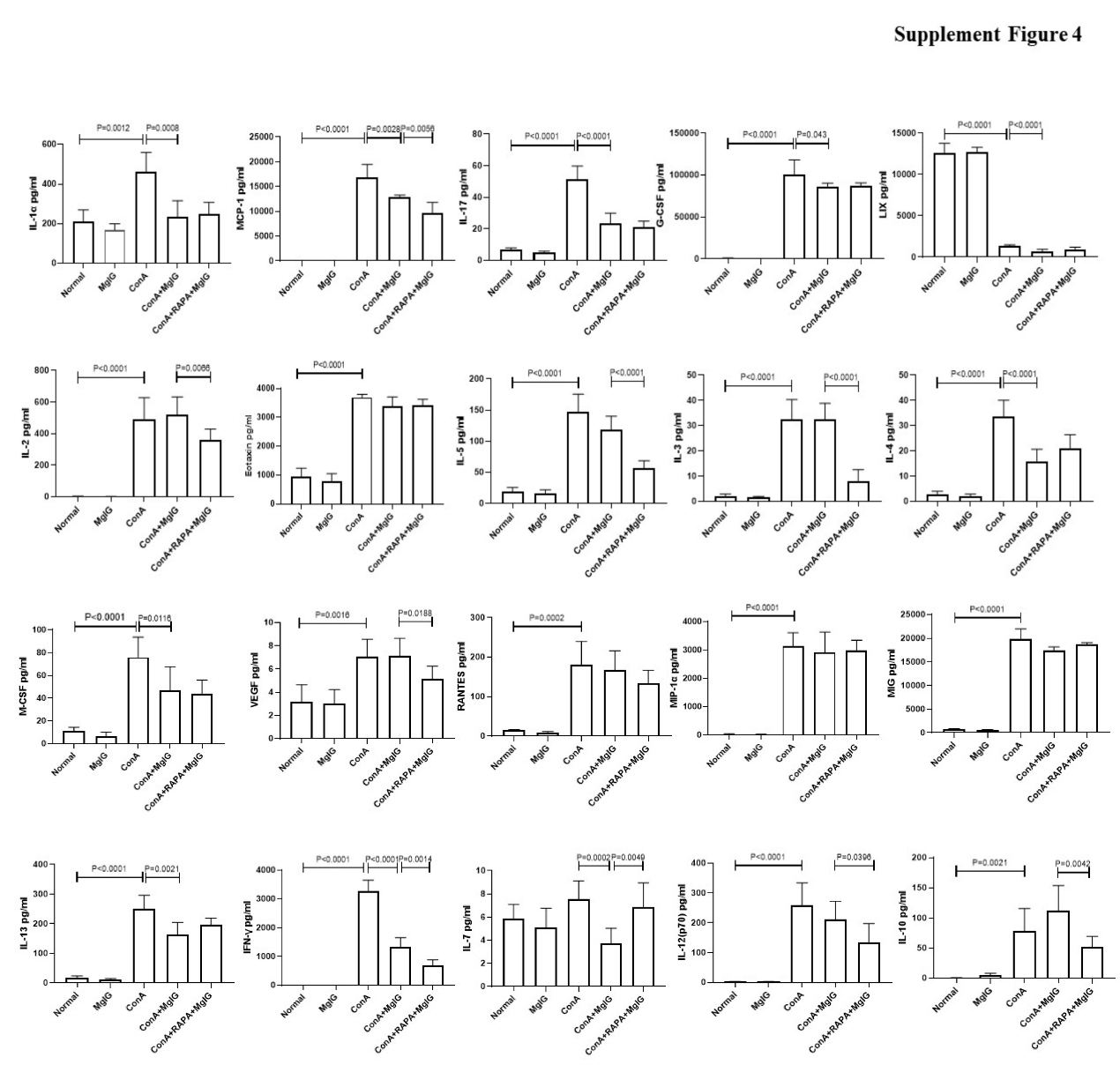


**S4. The expression levels of various cytokines in the serum in ConA-induced liver injury**. The data are shown as the mean and SD of at least three independent experiments. Cytokine levels in the sera of mice from each model group were measured with the Luminex Milliplex® MAP Kit.


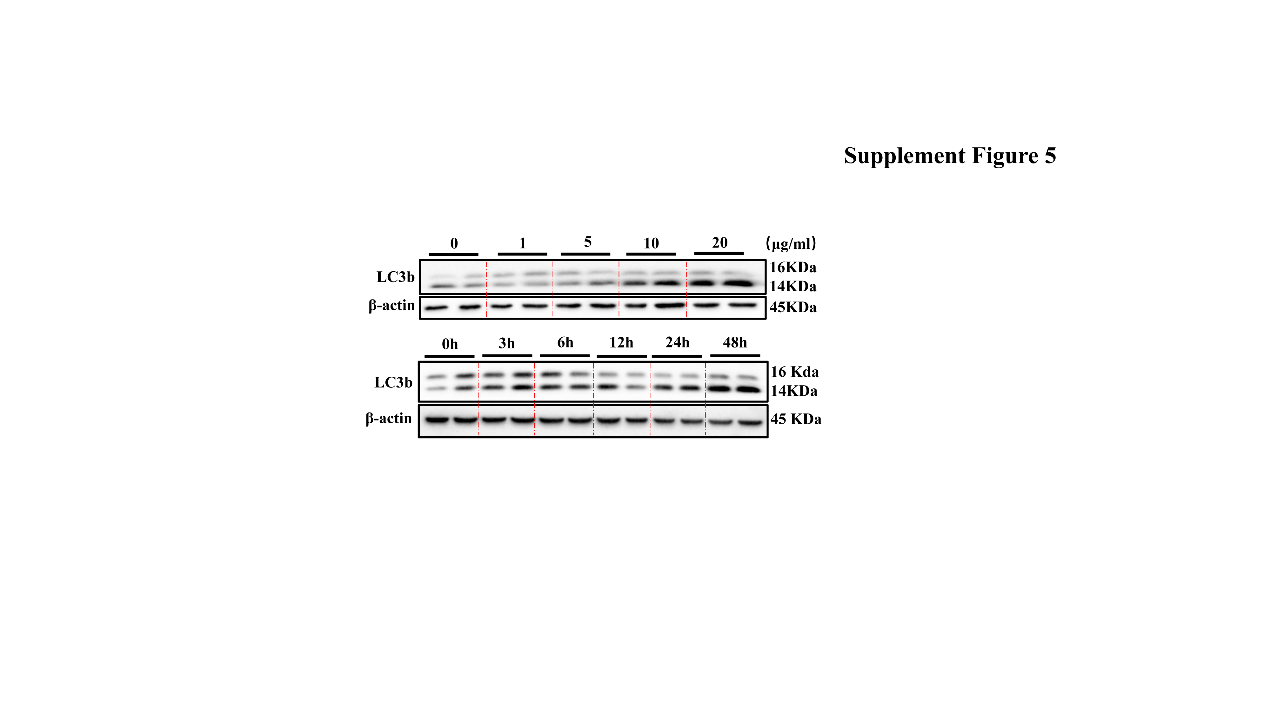


**S5. ConA induces autophagy in** **mouse primary hepatocytes.** At least three independent experiments were performed. The protein expression levels of LC3b in mouse primary hepatocytes treated with ConA (20 μg/ml) for different durations (0, 3, 6, 12, 24, or 48 h) and different concentrations of ConA (0, 1, 5, 10, or 20 μg/ml) for 24 h were measured by western blotting, with β-actin as a control.


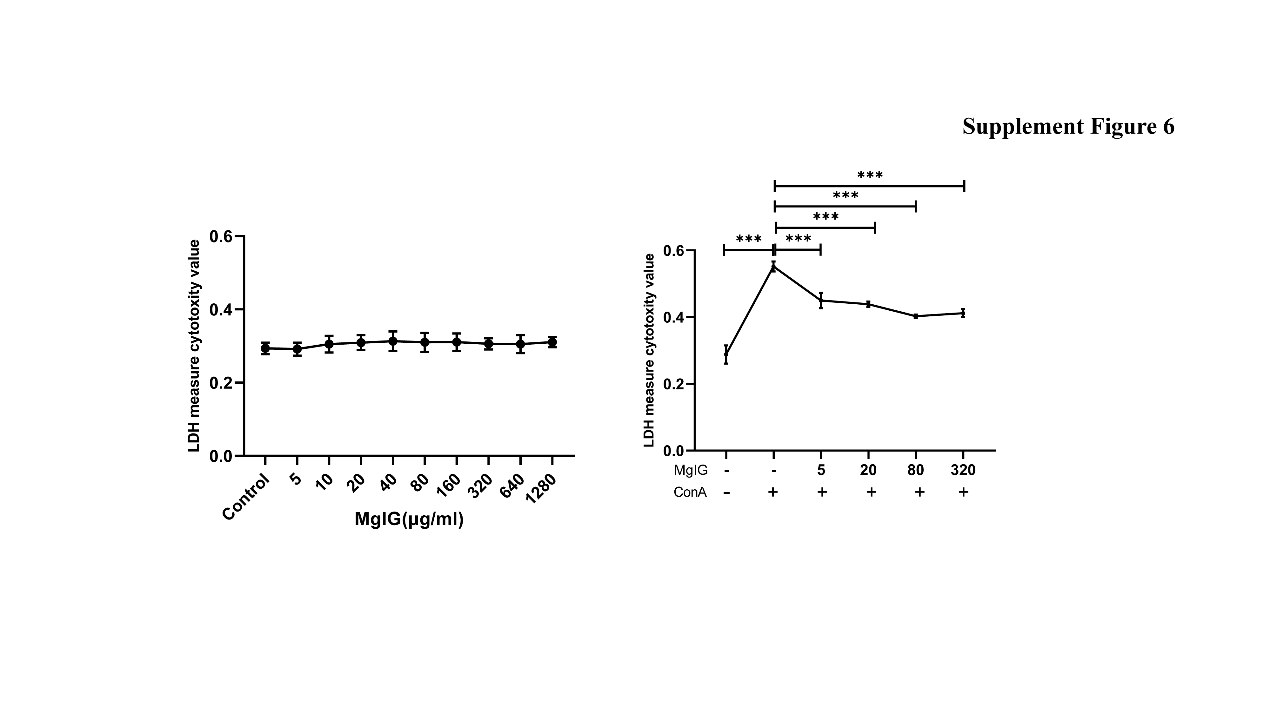


**S6.** **MgIG had no cytotoxicity effect on** **mouse primary hepatocytes and decreased hepatocyte cytotoxicity after ConA exposure.** Mouse primary hepatocytes were plated at a density of 1 × 10^4^ per well in a 96-well plate. Hepatocytes were treated with MgIG at different concentrations (0~1280μg/ml) for 1h. Additionally, primary hepatocytes were incubated with different concentrations of MgIG (5, 20, 80, or 320 μg/ml) for 1 h before being treated with ConA (20 μg/ml) for 24 h. Detection of the cytotoxicity was performed with an LDH cytotoxicity assay kit (Beyotime, Shanghai, China) according to the manufacturer’s instructions (***P<0.001).


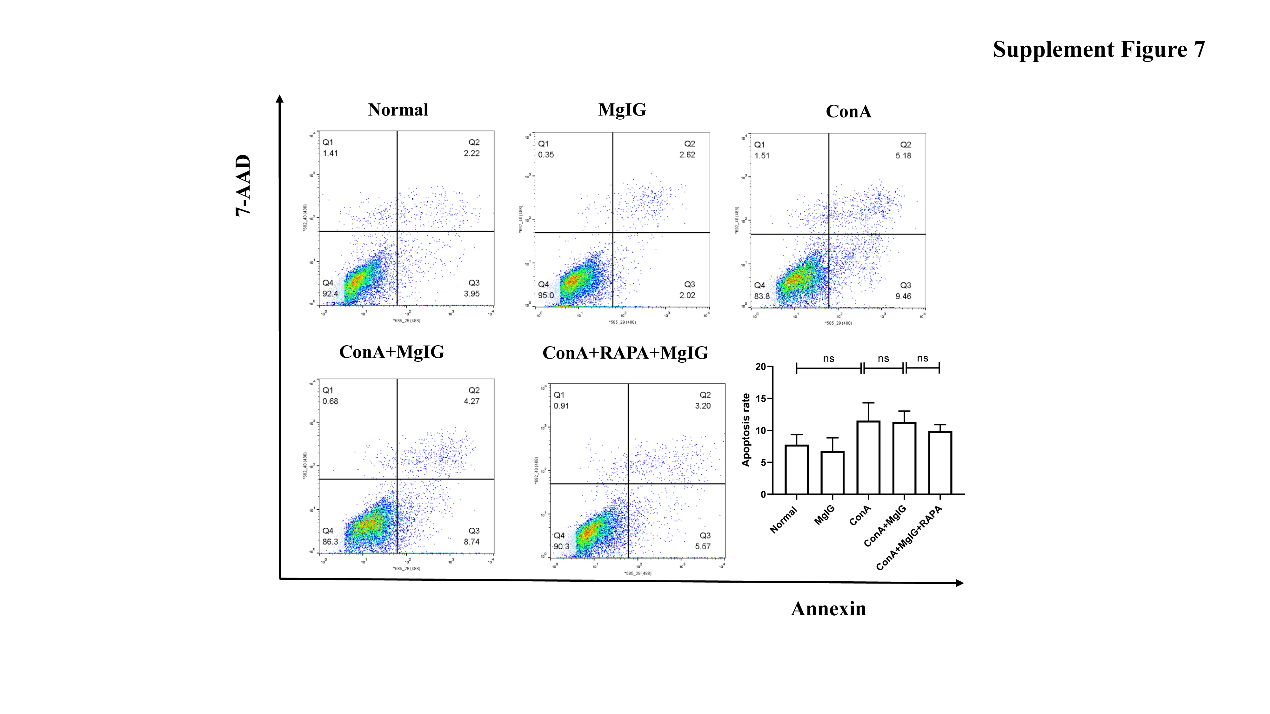


**S7. Evaluation of hepatocyte apoptosis induced by ConA in vitro by flow cytometry.** The data are shown as the mean SD of at least three independent experiments. Cell apoptosis was detected by Annexin V-phycoerythrin (PE)/7-amino-actinomycin (7-AAD) double staining (BD Bioscience, Franklin Lakes, NJ, USA). After treatment, the cells were resuspended in 1× binding buffer and then stained with PE and 7-AAD for 15 minutes in the dark. A FACScan flow cytometer (BD Bioscience) was used to analyze the samples, and the data were analyzed with FlowJo software.
